# Differences in spinal postures and mobility among adults with Prader-Willi syndrome, essential obesity, and normal-weight individuals

**DOI:** 10.3389/fendo.2023.1235030

**Published:** 2023-09-20

**Authors:** Munkh-Erdene Bayartai, Hannu Luomajoki, Gabriella Tringali, Roberta De Micheli, Graziano Grugni, Alessandro Sartorio

**Affiliations:** ^1^ Institute of Physiotherapy, School of Health Professions, Zurich University of Applied Sciences, (ZHAW), Winterthur, Switzerland; ^2^ Department of Physical and Occupational Therapy, School of Nursing, Mongolian National University of Medical Sciences, Ulaanbaatar, Mongolia; ^3^ Istituto Auxologico Italiano, Istituto di Ricovero e Cura a Carattere Scientifico (IRCCS), Experimental Laboratory for Auxo-Endocrinological Research, Piancavallo-Verbania, Italy; ^4^ Istituto Auxologico Italiano, Istituto di Ricovero e Cura a Carattere Scientifico (IRCCS), Division of Auxology, Piancavallo-Verbania, Italy

**Keywords:** Prader-Willi syndrome, syndromic obesity, adults, spinal posture, mobility

## Abstract

**Introduction:**

Spinal kinematics/motion are reported to be altered in adolescents and adults with essential obesity, while no information is available in patients with Prader-Willi syndrome so far. The aim of this study was to examine cross-sectionally the characteristics of spinal postures and mobility in 34 patients with PWS, in 35 age- and sex-matched adults with essential obesity, and in 37 normal-weight individuals.

**Methods:**

Spinal posture and mobility were assessed using a radiation-free back scan, the Idiag M360 (Idiag, Fehraltorf, Switzerland). Differences in spinal posture and mobility between the three groups were determined using a two-way analysis of variance.

**Results:**

Adults with Prader-Willi syndrome had greater thoracic kyphosis [difference between groups (Δ) = 9.6^0^, 95% CI 3.3^0^ to 15.6^0^, p = 0.001], less lumbar lordosis (Δ = -6.5^0^, 95% CI -12.7^0^ to -0.3^0^, p = 0.03) as well as smaller lumbar and hip mobility than those with normal weight.

**Discussion:**

Although the characteristics of the spine in patients with Prader-Will syndrome appear to be similar to that found in subjects with essential obesity, Prader-Willi syndrome was found to influence lumbar movements more than thoracic mobility. These results provide relevant information about the characteristics of the spine in adults with Prader-Willi syndrome to be taken into careful consideration in the management of spinal conditions. These findings also highlight the importance of considering the musculoskeletal assessment of spinal postures and approaches targeting spinal and hip flexibility in adults with Prader-Willi syndrome.

## Introduction

Prader-Willi syndrome (PWS) is a rare disease affecting approximately 1 in 21,000 newborns (1). It is considered the most common form of syndromic obesity and is caused by the failure of expression of paternally inherited genes in the PWS region of chromosome 15q11–13. The principal genetic mechanisms responsible for PWS are paternal deletion (DEL15) (60–70%), maternal uniparental disomy 15 (mUPD15) (25–35%), or imprinting defects and other alterations involving chromosome 15 (1–4%) (2).

PWS is characterized by a complex clinical condition including severe neonatal hypotonia and initial failure to thrive, followed by progressive hyperphagia with early childhood-onset obesity with its comorbidities unless food intake is strictly controlled. PWS also has characteristic dysmorphic features (characteristic facial appearance, small hands, and feet), behavioral problems, cognitive impairment, multiple endocrine abnormalities, and short stature for genetic background (3, 4).

Musculoskeletal manifestations are commonly found in PWS, including scoliosis, kyphosis, hip dysplasia, ligamentous laxity, and osteoporosis (5–7). Differently from non-syndromic obesity, patients with PWS showed an abnormal body composition characterized by a marked increase in fat mass associated with a decrease in lean mass, representing a unique congenital model of sarcopenia (8). Furthermore, although muscle hypotonia improves over time, adults with PWS remain mildly hypotonic (9). Overall, the presence of short stature, a high center of gravity, obesity, reduced muscle mass, and persistent muscle hypotonia make upright and plumb-line postural alignment more difficult.

From a biomechanical point of view, the combination of these abnormalities leads to impairment of motor and functional skills (10, 11). In adulthood, the most evident anomalies in motor control involve postural control (12), and gait (13). The aim of this study is to explore the characteristics of spinal postures and mobility in individuals with PWS, in age- and sex-matched adults with essential obesity, and in individuals with normal weight.

## Materials and methods

The study employed a cross-sectional design to explore the characteristics of spinal postures and mobility in individuals with PWS, comparing these results with those recorded in age- and sex-matched adults with essential obesity and in individuals with normal weight. The study has followed the “Strengthening the Reporting of Observational Studies in Epidemiology (STROBE)” recommendations (14).

### Participants

Individuals with PWS, essential obesity and normal weight were recruited into the present study. Normal weight and obesity were defined as a body mass index (BMI) < 25 and BMI >30, respectively, in accordance with the World Health Organization guidelines (15). All patients with PWS showed the typical clinical phenotype (3). Twenty-seven subjects had interstitial deletion of the proximal long arm of chromosome 15 (del15q11-q13), while 7 patients had uniparental maternal disomy for chromosome 15. Participants were excluded if they had past and present musculoskeletal or neurological conditions affecting spinal posture and mobility, such as limb length discrepancy, as well as those taking anti-inflammatory medications.

Individuals with PWS and essential obesity were hospitalized for a three-week multidisciplinary body weight reduction program at the Division of Auxology and at the Division of Metabolic Diseases, respectively, Istituto Auxologico Italiano, IRCCS, Piancavallo-Verbania, Italy, while subjects with normal weight were recruited from the general population in the Canton of Zurich, Switzerland. The Ethics Committee of Istituto Auxologico Italiano Milan, Italy (research code: 01C406; acronym: SCOLADUPWS; research project code: 01C124; acronym: PRORIPONATFIS) and the Ethics Committee of Zurich (BASEC-no. 2018-00979) approved the study. All procedures in the study were in compliance with the Helsinki Declaration of 1975, as revised in 2008. The research procedure was explained to each participant and written informed consent was obtained by subjects and their parents when it was appropriate. The sample size (n=106) had a power of 85% with an alpha of 0.05 to detect a medium effect (effect size = 0.3) for mean comparisons. Power analysis was performed using G-power 3.1 software (16).

### Measurements

A non-invasive, reliable, radiation-free, computer-aided skin-surface device, the Idiag M360 scan (Idiag, Fehraltorf, Switzerland) was used to assess spinal posture and mobility (17). The device is designed to quantify parameters related to the posture and mobility of the spine through computer-assisted analysis. The device records angles of each vertebral joint and sacral slope using computer-assisted analysis to quantify the spinal posture and mobility. During the recording, parameters including vertebral distances, positions of each vertebral bodies, angles created between them are determined. Vertebral distances and angles are measured whilst two rolling wheels embedded in the device follow the vertebral spinous processes during the measurement. The measured parameters, the data of which is sampled every 1.3 mm at a frequency of 150 Hz, are transferred radiographically to a personal computer through an analogue-digital converter as the rolling wheels follow the spinous processes (18, 19). Validity studies on measuring global and segmental lumbar motion using the Idiag M360, compared against an X-ray examination, considered the gold standard for determining spinal deformities (18, 20). Additionally, the reliability of the device has also been investigated in both normal-weight and obese individuals (21, 22). Intrarater reliability/intraclass correlation coefficients and standard error of measurement of the device for measurements of spinal postures in obese individuals ranged from 0.86 to 0.94 and 0.58^0^ to 0.70^0^, respectively. As regards spinal mobility, the intraclass correlation coefficients ranged from 0.57 to 0.80 and 0.87 to 0.98 in the frontal and sagittal planes (22). The intra and interrater reliability of the device for measurements of spinal posture and mobility in normal-weight individuals ranged from 0.61 to 0.96 and 0.70 to 0.93, respectively, whilst the standard error of measurement values ranged between 0.61^0^ and 13.18^0^ (21).

The spinal parameters as well as hip mobility were measured in the longitudinal and coronal planes as participants were instructed to bend forward (flexion), extension and then lateral flexion from an upright standing position, as described in the protocol of previous studies determining spinal posture and mobility (18, 21, 22). Both assessors who measured the spinal posture and mobility in patients with Prader-Willi syndrome and essential obesity, and in normal-weight individuals had been trained by the Idiag staff using the educational videos.

### Data processing

The difference between range of motion values in each segment of the spine measured at the standing position and the end of motion ranges in the sagittal and frontal planes was used to calculate each range of motion value for the spinal parameters. The sum of the respective range of motion values in each spinal segment (5 and 12 range of motion values for the lumbar and thoracic spine, respectively) were used to determine the total lumbar and thoracic range of motion of the spine. The sacral inclination in the sagittal plane was used to determine hip range of motion.

### Statistical analysis

Descriptive and inferential analyses were performed using the R version 4.2.2 (23). Mean values and standard deviations (SD) for participants’ age, sex, spinal posture and mobility, as well as hip motion and lumbar to hip ratio were determined in descriptive statistics. Shapiro–Wilk test was used to assess data normality. Differences in demographic and anthropometric characteristics between the three groups (Prader-Willi syndrome, essential obesity, normal weight) were determined using analysis of variance (ANOVA) for normally distributed data, the Kruskal Wallis test for non-normally distributed parameters and the Pearson’s chi-square test for categorical variables. Age and sex-adjusted two-way ANOVA was used to determine statistically significant differences in spinal posture and mobility between the three groups. Pairwise *post hoc* tests were applied using the software package “emmeans” to compare between groups following the ANOVA tests (24). Statistical significance was considered as a p value of less than 0.05.

## Results

**Figure 1:**
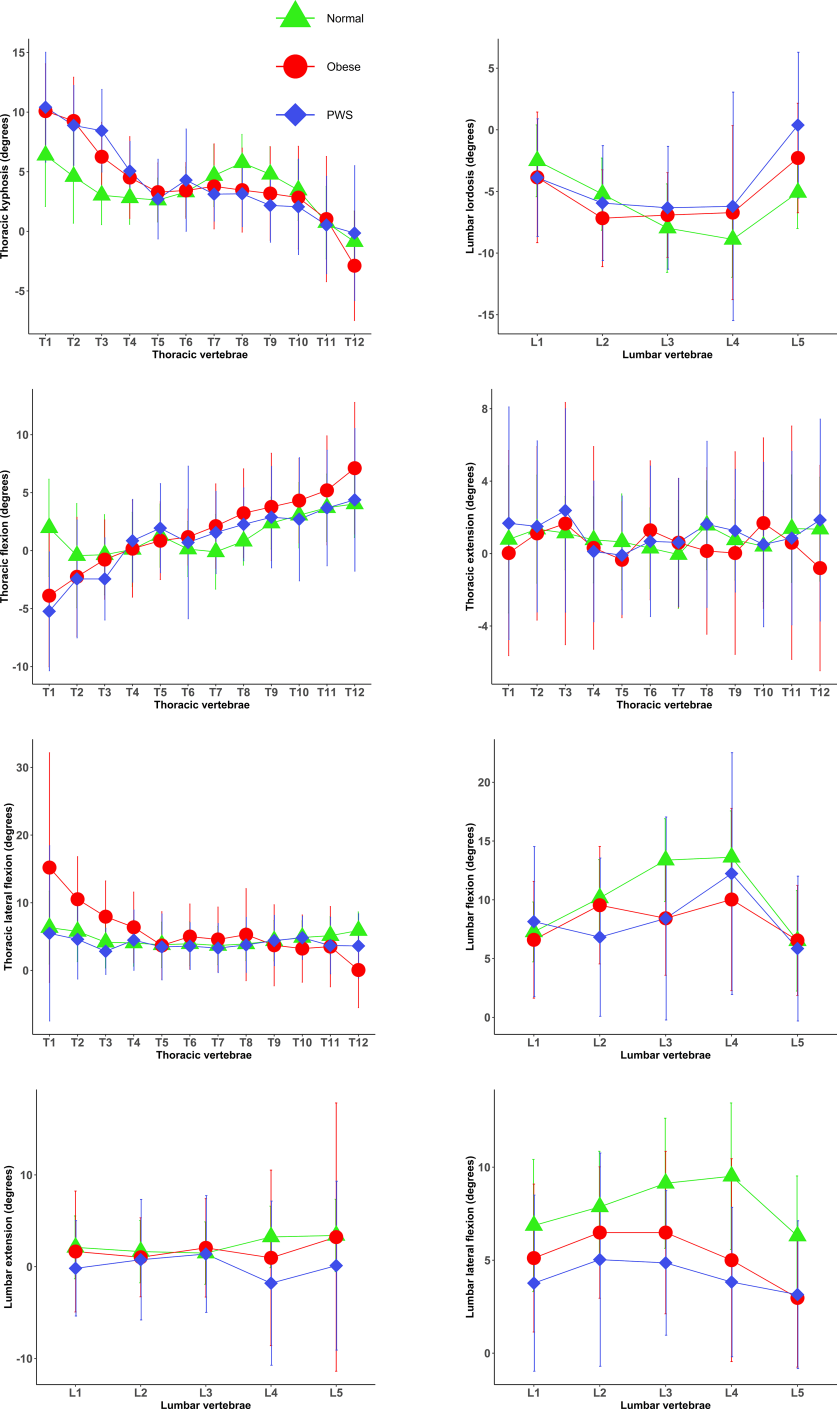
Posture and motion/kinematics of each individual spinal segment in the normal weight, obese and PWS groups (mean and standard deviations). Normal, normal-weight group; PWS, Prader–Willi syndrome group; Obese, essential obesity group.

A total of 37 normal-weight adults, 35 adults with essential obesity and 34 individuals with Prader-Willi syndrome were recruited into the study. Participants’ characteristics are presented in **Table 1**. No differences were observed in the mean age and sex ratio between the three groups (p>0.05). Spinal segmental posture and movements in the three groups of participants are illustrated in **Figure 1**.

**Table 1:** Main anthropometric characteristics of the study population, including normal-weight adults, adults with essential obesity, adults with Prader-Willi syndrome (mean ± standard deviations).

Variables	Normal-weight adults (N=37)	Adults with essential obesity (N=35)	Adults with Prader-Willi syndrome (N=34)	p value
Age (years)	39.4 (10.5)	41.4 (12.2)	35.9 (11.2)	^K^0.12
Sex (female, %)	76	74	59	^C^0.24
Weight (kg)	57.5 (7.2)	112.6 (17.8)	89.8 (20.7)	^K^<0.001*^”
Height (cm)	164.9 (8.0)	166.6 (9.3)	154.8 (2.8)	^K^<0.001^”
BMI (kg/m^2^)	21.1 (1.3)	40.3 (2.8)	37.6 (8.7)	^K^<0.001*^

p-value—statistical significance computed by using Kruskal Wallis test ^K^ and the chi-square test ^C^ for a comparison between the two groups.

* Normal-weight adults vs adults with essential obesity p< 0.05.

^ Normal-weight adults vs adults with Prader-Willi syndrome p< 0.05.

“ Adults with essential obesity vs adults with Prader-Willi syndrome p<0.05.

Spinal posture and mobility were not different in adults with Prader-Willi syndrome who were treated with recombinant growth hormone (rec-GH) during (6 females, 7 males) or before the study period (10 females, 11 males) compared to those with Prader-Willi syndrome never treated with rec-GH (p>0.05).

Almost all the spinal postures and movements measured were significantly different between adults with Prader-Willi syndrome and normal weight, except for sacral kyphosis, and thoracic mobility (**Table 2**). Adults with Prader-Willi syndrome had greater thoracic kyphosis but less lumbar lordosis and smaller movements in lumbar flexion, lumbar extension, lumbar lateral flexion and hip flexion as well as extension than those with normal weight. Thoracic flexion and lateral flexion were smaller in adults with Prader-Willi syndrome than in those with essential obesity, whereas thoracic extension was greater in those with Prader-Willi syndrome. Lumbar extension and lateral flexion were also smaller in patients with Prader-Willi syndrome compared to those with essential obesity but the remaining parameters measured were not different between these two groups (**Table 2**).

**Table 2:** Spinal postures, spinal and hip mobility in the three groups.

Variables	Normal weight adults (37) EMM (SE)	Adults with essential obesity (N=35) EMM (SE)	Adults with Prader-Willi syndrome (N=34) EMM (SE)	Differences in spinal posture and kinematics (95% CI)	p value
Spinal postures					
Thoracic Kyphosis (Th1-12)	40.9 (1.8)	47.9 (1.9)	50.5 (1.8) *	-9.6 (-15.9 to -3.3) *	0.001*
Proximal thoracic kyphosis (Th1-6)	22.0 (1.2)	36.1 (1.3)	39.6 (1.3) *	-17.6 (-21.9 to -13.3) *	< 0.001*
Distal thoracic kyphosis (Th7-12)	18.9 (1.7)	11.8 (1.7)	10.9 (1.7) *	8.0 (2.2 to 13.8) *	0.003*
Lumbar lordosis	27.6 (1.8)	25.1 (1.9)	21.1 (1.8) *	-6.5 (-12.7 to -0.3) *	0.03*
Sacral kyphosis	14.6 (1.5)	13.8 (1.6)	12.6 (1.6)	–	>0.05
Spinal mobility					
Thoracic (^0^)					
Flexion	16.8 (2.1)	21.0 (2.2)	11.4 (2.1) ^	9.6 (2.3 to 16.9) ^	0.005^
Extension	9.2 (1.7)	5.5 (1.8)	12.1 (1.8) ^	-6.5 (-12.6 to -0.4) ^	0.03^
Lateral flexion	55.9 (4.0)	68.8 (4.1)	48.4 (4.1) ^	20.4 (6.5 to 34.4) ^	0.001^
Lumbar (^0^)					
Flexion	50.3 (2.1)	41.1 (2.2)	40.3 (2.1) *	10.0 (2.6 to 16.4) *	0.003*
Extension	12.7 (2.1)	9.3	-0.2*^	12.9 (5.6 to 20.4) * 9.5 (1.9 to 17.1) ^	0.0001* 0.009^
Lateral flexion	39.5 (1.7)	26.6 (1.8)	19.4 (1.8) *^	20.1 (14.1 to 26.2) * 7.2 (1.1 to 13.4) ^	< 0.001* 0.01^
Hip mobility					
Hip (^0^)					
Flexion	61.4 (2.5)	42.8 (2.6)	37.4 (2.6) *	24.0 (15.3 to 32.7) *	< 0.001*
Extension	11.9 (2.5)	2.9 (2.6)	0.1 (2.5) *	11.8 (3.2 to 20.4) *	0.003*
Lumbar to hip ratio	0.42 (0.1)	0.44 (0.1)	0.37 (0.1)	–	>0.05

p-value (adjusted for age and sex)— the significance of differences between the groups. EMM estimated marginal means. SE standard errors. CI confidence interval. The lumbar to hip ratio was calculated by dividing lumbar range of motion by the sum of lumbar range of motion and hip range of motion during the trunk flexion in the sagittal plane.

* Adults with Prader-Willi syndrome vs normal-weight adults p< 0.05.

^ Adults with Prader-Willi syndrome vs adults with essential obesity p< 0.05.

## Discussion

The purpose of the present was to investigate spinal posture and mobility in patients with Prader-Willi syndrome, comparing the results with those found in age-, BMI- and sex-matched subjects with essential obesity and in normal-weight adults. The key finding was that almost all the spinal postures and movements measured were significantly different between adults with Prader-Willi syndrome and normal-weight individuals, except for sacral kyphosis and thoracic mobility, whereas only thoracic and lumbar movements were different between adults with Prader-Willi syndrome and those with essential obesity.

Individuals with Prader-Willi syndrome had greater thoracic kyphosis and less lumbar lordosis compared to normal-weight adults, while no significant differences were observed in spinal postures between adults with Prader-Willi syndrome and those with essential obesity. Consequences of alterations in spinal postures have been examined by previous studies in relation to musculoskeletal conditions (25, 26). For example, a previous case control study showed that increased thoracic kyphosis was associated with shoulder impingement syndrome in both individuals with and without shoulder impingement syndrome (25), highlighting the significance of taking thoracic kyphosis into account in the management of shoulder impingement syndrome. Additionally, increasing age appears to deepen alterations in thoracic kyphosis, as a systematic review reported that thoracic kyphosis increases by approximately 3 degrees per decade (27). Thoracic kyphosis also appears to change in the transition between sitting and standing positions (28, 29). For example, a prospective study found a decrease of 8.5 degrees in thoracic kyphosis in the transition from sitting to standing position (28), whilst a recent review study also reported that the thoracic kyphosis, lumbar lordosis and sacral slope reduced from standing to sitting by up to approximately 50% as sitting straightens the spine (29).

Although studies specifically exploring the influence of Prader-Willi syndrome on spinal posture and mobility are not available to date, few research works have explored obesity, which is one of the main features of subjects with Prader-Willi syndrome, in relation to alterations in spinal posture. For example, a previous cohort study of 1621 individuals with and without idiopathic scoliosis showed that obesity was associated with increased thoracic kyphosis in both participants with and without idiopathic scoliosis (26). Our previous study investigating the association of obesity with spinal posture in children and adolescents also showed that obesity was associated with increased thoracic kyphosis (22), implying that obesity may play an important role in the characteristics of spinal postures. In the present study, no significant differences in thoracic kyphosis were observed between adults with Prader-Willi syndrome and those with essential obesity, even if patients with Prader-Willi syndrome had greater thoracic kyphosis than normal-weight individuals. These results suggest that obesity, one of the main characteristics of Prader-Willi syndrome, appears to substantially contribute to increased thoracic kyphosis in those with Prader-Willi syndrome.

Sacral kyphosis was not different across the three groups, highlighting that neither obesity nor Prader-Willi syndrome is associated with alterations in sacral posture. Although previous studies did not find alterations in lumbar lordosis in subjects with essential obesity (22, 30, 31), our finding of a less lumbar lordosis in adult patients with Prader-Willi suggests the presence of specific alterations in spinal postures related to this clinical condition. Nevertheless, further research, particularly longitudinal studies could help to better understand the effect of Prader-Willi syndrome on the spine characteristics.

How adults with Prader-Willi move their spine was different compared to those with essential obesity and normal-weight individuals. Adults with Prader-Willi syndrome had smaller thoracic flexion, thoracic lateral flexion, lumbar extension and lumbar lateral flexion than those with essential obesity, whereas thoracic extension was greater in those with Prader-Willi syndrome. Lumbar movements measured were also smaller in patients with Prader-Willi syndrome compared to those with normal weight, while the thoracic mobility was not different between these two groups. Spinal mobility enables us to perform daily activities and its reduction is often associated with musculoskeletal conditions, particularly low back pain, implying the importance of preserving spinal mobility (32–34). For instance, a systematic review of prospective studies reported that reductions in lumbar mobility in the frontal plane predicted the development of low back pain (34). Additionally, previous studies examining the characteristics of individuals with obesity in comparison with that of normal-weight individuals reported that obesity was associated with reduced spinal flexibility (26, 35), suggesting that the influence of obesity on the characteristics of spinal mobility appears to be similar to that of Prader-Willi syndrome. Nevertheless, Prader-Willi syndrome appears to influence lumbar movements more than thoracic mobility, as the spinal mobility of adults with Prader-Willi syndrome was only different in lumbar movements compared to that of normal-weight individuals. These findings provide an important insight into the characteristics of spinal mobility of individuals with Prader-Willi syndrome.

Hip mobility was smaller in individuals with Prader-Willi syndrome than in normal-weight participants, but no differences in hip mobility were observed between individuals with obesity and those with Prader-Willi syndrome. Hip mobility also enables us to perform our daily activities and to move around in the environment. For example, some functional activities such as squatting, kneeling and cross-legged sitting require a large range of motion, allowing to conduct activities of daily living involving these functional activities (33). In addition, reduced hip mobility is linked with hip conditions such as groin pain (36). Exercises designed to increase hip flexibility appear to benefit those with non-specific low back pain in terms of pain and disability (37), showing the importance of hip flexibility in the management of musculoskeletal conditions associated with reduced hip mobility. Although studies examining the association between obesity and hip mobility are scarce, our previous study of 199 children/adolescents found that obesity was associated with reduced hip mobility, implying that hip mobility in adults with Prader-Willi appears to be similar to that recorded in adults with essential obesity. Lumbar to hip ratio was not different between normal-weight individuals and those with Prader-Willi syndrome, which could be explained by reduced flexibility observed in both the hip and lumbar movements as the ratio is defined by the proportion of these two variables. These findings suggest the importance of taking into account the characteristics of hip flexibility in the management of musculoskeletal conditions such as non-specific low back pain/groin pain in adults with Prader-Willi syndrome.

We acknowledge that the present study has several limitations. The present study employed the cross-sectional design, which precludes any causal interpretations of the observed association between Prader-Willi syndrome and spinal posture and mobility. The accuracy of the Idiag M360 as a skin surface device could potentially be affected by skin movement artefact, particularly in those with obesity for the measurement of spinal posture and movements. In the present study, spinal postures were assessed only in the sagittal plane since validity and reliability studies for measurements of spinal postures in the frontal plane in adults using the Idiag M360 are sparse. This approach prevented us to provide any information on the alterations in spinal curvatures in the frontal plane, such as scoliosis (which is common in individuals with Prader-Willi syndrome), and on the possible differences among the three groups. Although mean BMI was actually comparable between adults with Prader-Willi syndrome and those with essential obesity, average height and weight were significantly different between these two groups (due to the intrinsic anthropometric characteristics of the syndrome). These differences could potentially affect the comparison of spinal postures and mobility. Although BMI is actually one of the most popular ways to measure body composition as it pertains to health, body composition analysis by dual-energy x-ray absorptiometry (or bioimpedentiometry) would have given more reliable information on body composition. However, in the present study, we originally decided to avoid body composition measurements in the three subgroups since the measurements would have been done with different instruments and in two different Centers, potentially leading to errors in the measurements. This relevant aspect will however be taken into careful consideration in further future studies on this topic.

However, the study was strengthened by providing detailed information about the characteristics of the spine such as global as well as segmental posture and motion of the spine in adults with Prader-Willi syndrome and essential obesity as well as normal-weight controls. In addition, the relatively broad study population (Prader-Willi syndrome, essential obesity and normal-weight controls) was evaluated in the two research centers by well-trained staff in standardized experimental conditions.

Prader-Willi syndrome was associated with increased thoracic kyphosis, decreased lumbar lordosis and reduced spinal mobility in adults. Spinal posture and mobility were significantly different between adults with Prader-Willi syndrome and normal-weight individuals, except for sacral kyphosis and thoracic mobility, whereas only spinal mobility was different between adults with Prader-Willi syndrome and subjects with essential obesity. Although the characteristics of the spine in patients with Prader-Will syndrome appear to be similar to that found in age-, BMI- and sex-matched subjects with essential obesity, Prader-Willi syndrome was found to influence lumbar movements more than thoracic mobility. These results provide relevant important information about the characteristics of the spine in adults with Prader-Willi syndrome to be taken into careful consideration in the management of spinal conditions. These findings also highlight the importance of considering the musculoskeletal assessment of spinal postures and approaches targeting spinal and hip flexibility in adults with Prader-Willi syndrome.

## Data availability statement

Raw data are available upon a reasonable request to the corresponding author and they will be upoloaded on Zenodo.org immediately after the publication of the article.

## Ethics statement

The Ethics Committee of Istituto Auxologico Italiano Milan, Italy (research code: 01C406; acronym: SCOLADUPWS; research project code: 01C124; acronym: PRORIPONATFIS) and the Ethics Committee of Zurich (BASEC-no. 2018-00979) approved the study. The studies were conducted in accordance with the local legislation and institutional requirements. The participants provided their written informed consent to participate in this study.

## Author contributions

Conceptualization: AS, HL, and GG; Data acquisition: RM, GT, and LA; Formal analysis: M-EB; Funding acquisition: AS; Investigation: RM, GT, and LA; Supervision, AS, HL, and GG; Writing original draft: M-EB; Writing, review and editing: AS, HL, GG, and M-EB. All authors contributed to the article and approved the submitted version.

## References

[1] Bar C Diene G Molinas C Bieth E Casper C Tauber M. Early diagnosis and care is achieved but should be improved in infants with Prader-Willi syndrome. Orphanet J Rare Dis (2017) 12(1):118. doi: 10.1186/s13023-017-0673-6 28659150 PMC5490212

[2] Butler MG Hartin SN Hossain WA Manzardo AM Kimonis V Dykens E. Molecular genetic classification in Prader-Willi syndrome: a multisite cohort study. J Med Genet (2019) 56(3):149–53. doi: 10.1136/jmedgenet-2018-105301 PMC7387113 29730598

[3] Angulo MA Butler MG Cataletto ME. Prader-Willi syndrome: a review of clinical, genetic, and endocrine findings. J Endocrinol Invest (2015) 38(12):1249–63. doi: 10.1007/s40618-015-0312-9 PMC4630255 26062517

[4] Tauber M Hoybye C. Endocrine disorders in Prader-Willi syndrome: a model to understand and treat hypothalamic dysfunction. Lancet Diabetes Endocrinol (2021) 9(4):235–46. doi: 10.1016/S2213-8587(21)00002-4 33647242

[5] Shim JS Lee SH Seo SW Koo KH Jin DK. The musculoskeletal manifestations of Prader-Willi syndrome. J Pediatr Orthop (2010) 30(4):390–5. doi: 10.1097/BPO.0b013e3181da857d 20502241

[6] Crinò A Armando M Crostelli M Mazza O Bruzzese D Convertino A. High prevalence of scoliosis in a large cohort of patients with Prader-Willi syndrome. J Clin Med (2022) 11(6). doi: 10.3390/jcm11061574 PMC8953215 35329900

[7] van Abswoude DH Pellikaan K Rosenberg AGW Davidse K Coupaye M Høybye C. Bone health in adults with Prader-Willi syndrome: clinical recommendations based on a multicenter cohort study. J Clin Endocrinol Metab (2022) 108(1):59–84. doi: 10.1210/clinem/dgac556 36149817 PMC9759176

[8] Irizarry KA Miller M Freemark M Haqq AM. Prader Willi syndrome: genetics, metabolomics, hormonal function, and new approaches to therapy. Adv Pediatr (2016) 63(1):47–77. doi: 10.1016/j.yapd.2016.04.005 27426895 PMC4955809

[9] Cassidy SB Schwartz S Miller JL Driscoll DJ. Prader-Willi syndrome. Genet Med (2012) 14(1):10–26. doi: 10.1038/gim.0b013e31822bead0 22237428

[10] Reus L Zwarts M van Vlimmeren LA Willemsen MA Otten BJ Nijhuis-van der Sanden MW. Motor problems in Prader-Willi syndrome: a systematic review on body composition and neuromuscular functioning. Neurosci Biobehav Rev (2011) 35(3):956–69. doi: 10.1016/j.neubiorev.2010.10.015 21056055

[11] Belluscio V Bergamini E Salatino G Marro T Gentili P Iosa M. Dynamic balance assessment during gait in children with Down and Prader-Willi syndromes using inertial sensors. Hum Mov Sci (2019) 63:53–61. doi: 10.1016/j.humov.2018.11.010 30503982

[12] Capodaglio P Menegoni F Vismara L Cimolin V Grugni G Galli M. Characterisation of balance capacity in Prader-Willi patients. Res Dev Disabil (2011) 32(1):81–6. doi: 10.1016/j.ridd.2010.09.002 20884170

[13] Cimolin V Cau N Galli M Pau M Parisio C Saezza A. Gait strategy and body composition in patients with Prader-Willi syndrome. Eat Weight Disord (2021) 26(1):115–24. doi: 10.1007/s40519-019-00825-2 31797332

[14] von Elm E Altman DG Egger M Pocock SJ Gøtzsche PC Vandenbroucke JP. The Strengthening the Reporting of Observational Studies in Epidemiology (STROBE) statement: guidelines for reporting observational studies. J Clin Epidemiol (2008) 61(4):344–9. doi: 10.1016/j.jclinepi.2007.11.008 18313558

[15] World Health Organization. Obesity (2023). Available at: https://www.who.int/tools/growth-reference-data-for-5to19-years/indicators/bmi-for-age.

[16] Faul F Erdfelder E Buchner A Lang A-G. Statistical power analyses using G*Power 3.1: Tests for correlation and regression analyses. Behav Res Methods (2009) 41(4):1149–60. doi: 10.3758/BRM.41.4.1149 19897823

[17] Idiag. Idiag M360 (2021). Available at: https://www.idiag.ch/en/idiag-m360-en/.

[18] Livanelioglu A Kaya F Nabiyev V Demirkiran G Fırat T. The validity and reliability of "Spinal Mouse" assessment of spinal curvatures in the frontal plane in pediatric adolescent idiopathic thoraco-lumbar curves. Eur Spine J (2016) 25(2):476–82. doi: 10.1007/s00586-015-3945-7 25900295

[19] Mannion AF Knecht K Balaban G Dvorak J Grob D. A new skin-surface device for measuring the curvature and global and segmental ranges of motion of the spine: reliability of measurements and comparison with data reviewed from the literature. Eur Spine J (2004) 13(2):122–36. doi: 10.1007/s00586-003-0618-8 PMC3476568 14661104

[20] Nouh MR. Imaging of the spine: Where do we stand? World J Radiol (2019) 11(4):55–61. doi: 10.4329/wjr.v11.i4.55 31110605 PMC6503457

[21] Kellis E Adamou G Tzilios G Emmanouilidou M. Reliability of spinal range of motion in healthy boys using a skin-surface device. J Manipulative Physiol Ther (2008) 31(8):570–6. doi: 10.1016/j.jmpt.2008.09.001 18984239

[22] Bayartai ME Schaer CE Luomajoki H Tringali G De Micheli R Sartorio A. Differences in spinal posture and mobility between children/adolescents with obesity and age-matched normal-weight individuals. Sci Rep (2022) 12(1). doi: 10.1038/s41598-022-19823-z PMC9481592 36114222

[23] Core Team R. R: A language and environment for statistical computing. Vienna, Austria: R Foundation for Statistical Computing (2019).

[24] Lenth R. Lenth R. emmeans: Estimated Marginal Means, aka Least-Squares Means. R package emmeans version 1.7.0. (2021).

[25] Hunter DJ Rivett DA McKeirnan S Smith L Snodgrass SJ. Relationship between shoulder impingement syndrome and thoracic posture. Phys Ther (2020) 100(4):677–86. doi: 10.1093/ptj/pzz182 31825488

[26] Valdovino AG Bastrom TP Reighard FG Cross M Bartley CE Shah SA. Obesity is associated with increased thoracic kyphosis in adolescent idiopathic scoliosis patients and nonscoliotic adolescents. Spine Deform (2019) 7(6):865–9. doi: 10.1016/j.jspd.2019.03.010 31731995

[27] Pan F Firouzabadi A Reitmaier S Zander T Schmidt H. The shape and mobility of the thoracic spine in asymptomatic adults - A systematic review of in *vivo* studies. J Biomech (2018) 78:21–35. doi: 10.1016/j.jbiomech.2018.07.041 30100219

[28] Hey HWD Teo AQA Tan K-A Ng LWN Lau L-L Liu K-PG. How the spine differs in standing and in sitting—important considerations for correction of spinal deformity. Spine J (2017) 17(6):799–806. doi: 10.1016/j.spinee.2016.03.056 27063999

[29] Tsagkaris C Widmer J Wanivenhaus F Redaelli A Lamartina C Farshad M. The sitting vs standing spine. North Am Spine Soc J (NASSJ) (2022) 9:100108. doi: 10.1016/j.xnsj.2022.100108 PMC8924684 35310424

[30] Canbek U Rosberg DBH Rosberg HE Canbek TD Akgün U Comert A. The effect of age, BMI, and bone mineral density on the various lumbar vertebral measurements in females. Surg Radiol Anat (2021) 43(1):101–8. doi: 10.1007/s00276-020-02560-1 32876743

[31] Romero-Vargas S Zárate-Kalfópulos B Otero-Cámara E Rosales-Olivarez L Alpízar-Aguirre A Morales-Hernández E. The impact of body mass index and central obesity on the spino-pelvic parameters: a correlation study. Eur Spine J (2013) 22(4):878–82. doi: 10.1007/s00586-012-2560-0 PMC3631046 23149493

[32] Bible JE Biswas D Miller CP Whang PG Grauer JN. Normal functional range of motion of the lumbar spine during 15 activities of daily living. J Spinal Disord Tech (2010) 23(2):106–12. doi: 10.1097/BSD.0b013e3181981823 20065869

[33] Hemmerich A Brown H Smith S Marthandam SS Wyss UP. Hip, knee, and ankle kinematics of high range of motion activities of daily living. J Orthopaedic Res (2006) 24(4):770–81. doi: 10.1002/jor.20114 16514664

[34] Sadler SG Spink MJ Ho A De Jonge XJ Chuter VH. Restriction in lateral bending range of motion, lumbar lordosis, and hamstring flexibility predicts the development of low back pain: a systematic review of prospective cohort studies. BMC Musculoskeletal Disord (2017) 18(1):179–. doi: 10.1186/s12891-017-1534-0 PMC5418732 28476110

[35] Gilleard W Smith T. Effect of obesity on posture and hip joint moments during a standing task, and trunk forward flexion motion. Int J Obes (2005) (2007) 31(2):267–71. doi: 10.1038/sj.ijo.0803430 16801923

[36] Tak I Engelaar L Gouttebarge V Barendrecht M Van den Heuvel S Kerkhoffs G. Is lower hip range of motion a risk factor for groin pain in athletes? A systematic review with clinical applications. Br J Sports Med (2017) 51(22):1611–21. doi: 10.1136/bjsports-2016-096619 PMC5754850 28432076

[37] Hatefi M Babakhani F Ashrafizadeh M. The effect of static stretching exercises on hip range of motion, pain, and disability in patients with non-specific low back pain. J Exp Orthop (2021) 8(1):55. doi: 10.1186/s40634-021-00371-w 34318348 PMC8316530

